# Effect of dietary prebiotic supplementation on advanced glycation, insulin resistance and inflammatory biomarkers in adults with pre-diabetes: a study protocol for a double-blind placebo-controlled randomised crossover clinical trial

**DOI:** 10.1186/1472-6823-14-55

**Published:** 2014-07-10

**Authors:** Nicole J Kellow, Melinda T Coughlan, Gayle S Savige, Christopher M Reid

**Affiliations:** 1 Department of Epidemiology & Preventive Medicine, School of Public Health & Preventive Medicine, Monash University, The Alfred Centre, Melbourne, Victoria 3004, Australia; 2 Glycation, Nutrition & Metabolism Laboratory, Baker IDI Heart & Diabetes Institute, Melbourne, Victoria 8008, Australia; 3 Department of Medicine, Central Clinical School, Monash University, Alfred Medical Research & Education Precinct, Melbourne, Victoria 3004, Australia

**Keywords:** Advanced glycation end products, Maillard reaction, Prebiotics, Gut microbiota, Type 2 diabetes mellitus, Insulin resistance, Inflammation

## Abstract

**Background:**

Advanced glycation endproducts (AGEs) contribute to the development of vascular complications of diabetes and have been recently implicated in the pathogenesis of diabetes. Since AGEs are generated within foodstuffs upon food processing, it is increasingly recognised that the modern diet is replete with AGEs. AGEs are thought to stimulate chronic low-grade inflammation and promote oxidative stress and have been linked to the development of insulin resistance. Simple therapeutic strategies targeted at attenuating the progression of chronic low-grade inflammation and insulin resistance are urgently required to prevent or slow the development of type 2 diabetes in susceptible individuals. Dietary modulation of the human colonic microbiota has been shown to confer a number of health benefits to the host, but its effect on advanced glycation is unknown. The aim of this article is to describe the methodology of a double-blind placebo-controlled randomised crossover trial designed to determine the effect of 12 week consumption of a prebiotic dietary supplement on the advanced glycation pathway, insulin sensitivity and chronic low-grade inflammation in adults with pre-diabetes.

**Methods/Design:**

Thirty adults with pre-diabetes (Impaired Glucose Tolerance or Impaired Fasting Glucose) aged between 40–60 years will be randomly assigned to receive either 10 grams of prebiotic (inulin/oligofructose) daily or 10 grams placebo (maltodextrin) daily for 12 weeks. After a 2-week washout period, study subjects will crossover to receive the alternative dietary treatment for 12 weeks. The primary outcome is the difference in markers of the advanced glycation pathway carboxymethyllysine (CML) and methylglyoxal (MG) between experimental and control treatments. Secondary outcomes include HbA_1c_, insulin sensitivity, lipid levels, blood pressure, serum glutathione, adiponectin, IL-6, E-selectin, myeloperoxidase, C-reactive protein, Toll-like Receptor 4 (TLR4), soluble receptor for AGE (sRAGE), urinary 8-isoprostanes, faecal bacterial composition and short chain fatty acid profile. Anthropometric measures including BMI and waist circumference will be collected in addition to comprehensive dietary and lifestyle data.

**Discussion:**

Prebiotics which selectively stimulate the growth of beneficial bacteria in the human colon might offer protection against AGE-related pathology in people at risk of developing type 2 diabetes.

**Trial registration:**

Australia and New Zealand Clinical Trials Register (ANZCTR): ACTRN12613000130763.

## Background

Advanced Glycation Endproducts (AGEs) are formed via the Maillard reaction, which consists of a complex network of non-enzymatic reactions involving the carbonyl groups of reducing sugars which react with the amino groups of proteins [1]. AGEs are generated *in vivo* as a normal consequence of metabolism, but their formation is accelerated under conditions where blood glucose is chronically elevated such as poorly controlled diabetes [2]. AGE formation is also increased in the presence of oxidative stress, which is frequently observed in individuals with the metabolic syndrome [3,4]. Overproduction of reactive oxygen species (ROS) can result in maladaptive responses including interruption of cellular glycolysis, which can generate highly reactive dicarbonyl compounds capable of rapid AGE formation [5].

Excessive AGE accumulation can lead to several pathophysiological consequences. AGE-modification of proteins results in changes in structure and/or function. For example, the AGE-modification of extracellular collagen reduces its elasticity and solubility, and results in increased stiffness, disturbed cellular adhesion and reduced turnover contributing to basement membrane thickening [6]. Intracellularly, AGE-modification of mitochondrial proteins is associated with suppression in the activity of respiratory chain enzymes and overproduction of ROS [7,8]. Indeed, glycated proteins provide stable active sites for catalysing the formation of free radicals [9]. Finally, AGEs are able to bind and activate a range of receptors, which then trigger a downstream cascade of pathogenic mediators. Interaction of AGEs with the Receptor for AGEs (RAGE) promotes activation of the transcription factor nuclear factor kappa-B (NF-κB), with subsequent upregulation of chemokines, such as MCP-1 and profibrogenic mediators such as TGFβ in addition to pro-inflammatory cytokines which are known to be involved in thrombogenesis, vascular inflammation and pathological angiogenesis. These RAGE-mediated events contribute to many of the long-term complications of diabetes [10]. AGE/RAGE ligation also promotes overproduction of ROS which can then activate NF-κB [11], a key driver of inflammation.

More recently, AGEs have been implicated in the pathogenesis of both type 1 and type 2 diabetes. Several studies have shown that AGEs are associated with insulin resistance [12,13], and can induce low-grade inflammation [14] and pancreatic beta cell dysfunction [15,16].

In contrast to endogenous AGE formation, AGEs are also absorbed by the body from exogenous sources such as cigarette smoke and through consumption of processed foods [17]. Since AGEs are generated within foodstuffs upon heating and food processing, it is increasingly recognised that the modern diet is replete with AGEs [18]. AGE-restricted diets can arrest the development of type 2 diabetes in animal models [19]. A recent study found that excess consumption of AGE-precursors in mice over several generations led to the development of insulin resistance [20]. Human trials have found that dietary AGE restriction can improve insulin sensitivity [21,22] and decrease markers of oxidative stress [23] or inflammation [24]. Further studies are required to confirm the long-term benefits of dietary AGE-restriction in humans [25]. However, simple, safe and effective interventions which prevent or minimise excessive AGE accumulation and subsequent AGE-related pathology in people with diabetes and/or in those at risk of developing the condition are warranted.

Interventions which influence the human intestinal microbiota are worthy of further investigation given that specific micro-organisms have the ability to significantly affect host metabolism. Gut bacteria play an important role in the host immune system, modulation of inflammatory processes, extraction of energy from the host’s diet, fermentation of dietary fibres to produce short-chain fatty acids, alteration of human gene expression, regulation of intestinal permeability, production of some vitamins and promotion of mineral absorption by the host [26-31]. Furthermore, the total quantity and relative proportions of distinct bacterial species found in the colon differ between lean and obese individuals as well as between individuals with and without diabetes [32,33].

It is thought that certain dietary AGEs are largely undigested by human gut enzymes and eventually enter the colon, where they may act as a growth substrate for detrimental bacteria such as some Clostridium and Bacteroides species [34]. Therefore it is conceivable that individuals who consume highly processed diets (which contain large quantities of AGEs) may adversely alter their colonic microbial composition, potentially enhancing their risk for the development of metabolic diseases such as obesity and type 2 diabetes [35].

Therapeutic manipulation of the gut microbiota and restoration of normobiosis could potentially reduce circulating AGE levels and improve the metabolic health of individuals at risk for the development of type 2 diabetes. Regular consumption of prebiotics to promote the growth of beneficial gut bacterial flora is one such avenue currently under investigation. Prebiotics are non-digestable plant-derived carbohydrates which confer health benefits to the host by acting as a fermentation substrate in the colon, stimulating the preferential growth and activity of a limited number of beneficial microbial species [36]. Supplementation of the human diet with prebiotic fructans such as inulin or fructo-oligosaccharides alters the bacterial composition of the large intestine by favouring the selective proliferation of beneficial lactic acid-producing species such as bifidobacteria and lactobacilli. Prebiotic-stimulated increases in intestinal Bifidobacterium species have been shown to attenuate the production of ROS and markers of inflammation in individuals consuming high fat diets [37].

While the complex interactions between diet, intestinal microbiota and host metabolism are still being elucidated, no studies have investigated the effect of dietary prebiotics on circulating AGE concentrations. This trial was designed to investigate the effect of a prebiotic dietary supplement on AGE accumulation and explore changes to the growth and activity of specific gut microbiota in adults diagnosed with prediabetes.

## Methods/Design

### Study design and setting

**Figure 1:**
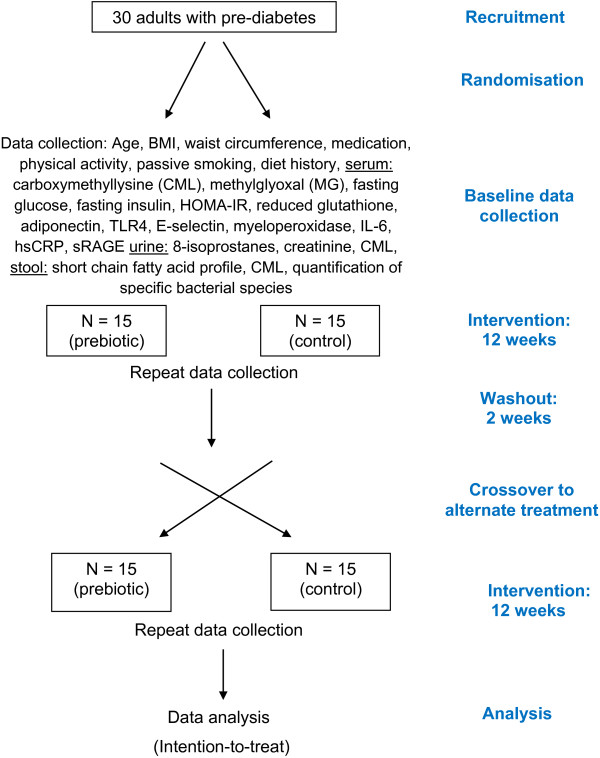
Trial protocol.

This is a 6.5-month randomised crossover controlled clinical trial (RCT) in which adults aged 40–60 years with diagnosed pre-diabetes will be enrolled. Potential study participants will be identified from General Practice (GP) clinics throughout South Gippsland, Victoria. The study design is presented in Figure 1.

### Inclusion criteria

Individuals aged between 40–60 years and diagnosed with prediabetes (Impaired Fasting Glucose or Impaired Glucose Tolerance) within the previous 12 months. Diagnosis will have been made at each individual’s local GP clinic after undertaking an Oral Glucose Tolerance Test (OGTT). Prediabetes was defined as a fasting plasma glucose concentration ≥ 6.1 and < 7.0 mmol/L followed by a 2-hour post glucose load glucose concentration < 7.8 mmol/L, or a fasting plasma glucose < 7.0 mmol/L followed by a 2-hour post glucose load glucose concentration ≥ 7.8 and < 11.1 mmol/L [38].

### Exclusion criteria

Individuals previously diagnosed with type 1, type 2 diabetes or impaired renal function (eGFR <90 mL/min/1.73 m^2^), individuals with known gastrointestinal pathology (coeliac disease, inflammatory bowel disease), pregnant women, smokers, individuals who have taken antibiotics, dietary prebiotic or probiotic nutritional supplements within the previous three months, individuals taking aspirin or Vitamin B, individuals who have made major dietary or lifestyle changes in the previous three months, individuals who are unwilling to provide blood, urine and stool samples or are unable to attend their local pathology collection centre.

### Ethics

The trial has received ethical approval from the Monash University Human Research Ethics Committee.

### Sample size calculation

The minimum difference we wish to detect is 0.4 micromol/L serum CML (20% reduction in CML), with a standard deviation of 0.4 [39] with 5% Significance and 80% Power. This calculates as a total sample size of 18 individuals, plus 12 individuals to allow for withdrawals = 30 subjects required. Epidemiological studies have demonstrated a positive correlation between serum CML and all-cause and cardiovascular mortality, cardiovascular disease, glucose intolerance, impaired insulin secretion, renal impairment and diabetic vascular complications [40,41]. A 0.4 micromol/L increase in serum CML concentration represented a 68% increased risk for all-cause mortality over seven years in a large prospective cohort study [42].

### Baseline assessment

**Figure 2:**
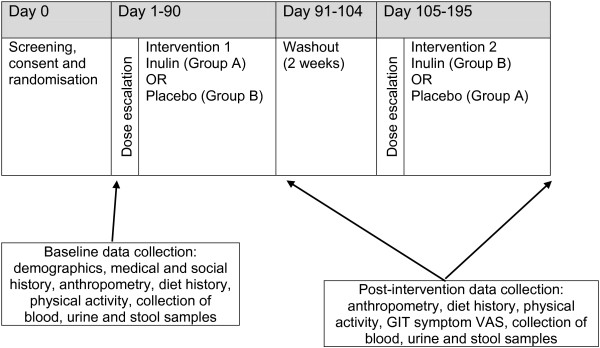
Study flowchart.

The study timeline is presented in Figure 2. Following recruitment and screening, consenting participants will undergo a baseline assessment at their local GP clinic. The baseline assessment will be undertaken by the research dietitian, who will collect demographic details, medical and social history (living situation, marital status, current occupation), physical activity questionnaire, dietary intake assessment, anthropometric measurements including Body Mass Index (BMI) and waist circumference. Participants will also be instructed to attend the local pathology centre to provide a stool sample, 24-hour urine collection, and have blood taken for analysis.

### Randomisation

Randomisation of participants to intervention/placebo sequence will be completed by a local pharmacist using a permuted-block randomisation stratified by gender via the web site http://www.randomization.com. The pharmacist will distribute the experimental and placebo nutritional supplements to participants in the appropriate sequence. Other than provision of supplements, the pharmacist will have no contact with study participants or involvement in data collection or analysis. Dietary supplements will be provided in sealed opaque packages which will be weighed at the conclusion of the study in order to assess compliance. The supplements will be packaged off-site by an external pharmaceutical packaging agency. Each package will contain a participant’s study identification number and will be labelled as either Supplement 1 or Supplement 2 corresponding to the first and second intervention periods. This will ensure all investigators and participants are blinded to the treatment. Blinding will cease only after statistical analysis of the data has been completed.

### Intervention

Participants will be randomly assigned to receive either 10 g of chicory-derived inulin/oligofructose powder (kindly provided by Beneo-Orafti Active Food Ingredients, Belgium) (intervention) or 10 g of maltodextrin powder (placebo) daily. Previous studies have demonstrated the bifidogenic effect of daily consumption of 5 g and 8 g dietary inulin supplements [43]. The inulin and maltodextrin powders to be consumed are both tasteless and can be mixed into hot or cold liquids or semi-solid foods. Participants will be instructed how to incorporate their supplement into their usual diet, and advised to gradually increase their dose over ten successive days until the target dose is reached. This stepped escalation in supplement dose aims to minimise gastrointestinal discomfort for participants, as a sudden increase in dietary prebiotic intake may result in increased stool frequency, abdominal bloating and flatulence until the bowel adapts to the increased fibre intake [44]. Written instructions will also be provided. Participants will be advised to consume each dietary supplement daily for 90 days, and otherwise maintain their usual dietary intake and level of physical activity. Gastrointestinal tolerance to the dietary supplements will be indicated by each study participant using a visual analogue scale as described below.

### Follow up visits

Visits to the GP clinic will be scheduled for each participant at the conclusion of both Intervention 1 and Intervention 2 treatment periods, in order to provide follow-up data. Information collected will include anthropometric measurements, dietary intake assessment and physical activity assessment. Each participant will complete a self-administered questionnaire designed to assess gastrointestinal tolerance to the dietary supplement. Completed questionnaires will be placed into sealed opaque envelopes in order to maintain blinding of the researcher collecting data during the follow-up visits. Participants will also be instructed to attend the local pathology centre to provide urine and stool samples, and have blood taken for analysis.

### Safety considerations

All adverse events will be documented.

### Outcome measurements

The primary outcome of interest is the difference in serum AGE and AGE-precursor concentration (measured as CML and MG respectively) between experimental and control treatments. Secondary outcomes include HbA1c, insulin resistance (measured indirectly by homeostasis model assessment), antioxidant capacity (reduced glutathione), markers of oxidative stress on lipid molecules (urinary 8-isoprostanes), inflammatory biomarkers (serum IL-6, high sensitivity C-reactive protein, MCP-1, sRAGE), adhesion molecules (E-selectin), gut barrier integrity (TLR4), 24-hour urine and faecal CML and MG concentrations, faecal bacterial composition (*Bifidobacterium* spp., *Lactobacillus* spp., *Roseburia* spp., *Faecalibacterium prausnitzii* and *Akkermansia muciniphila*) and faecal short chain fatty acid concentrations. Lipid levels (serum total cholesterol, LDL, HDL, TG) and blood pressure will also be measured. Anthropometric measurements including BMI and waist circumference will be collected, in addition to comprehensive dietary and lifestyle data. Gastrointestinal side-effects will be assessed using a visual analogue scale adapted from Lecerf *et al.*[45]. The scale rates nine items including flatulence, bloating, rumbling, abdominal cramps, a global digestive tolerance score calculated from the five previous items, stool consistency, stool frequency and general well-being through disturbances in usual and professional activities as well as disturbances in personal and social activities. Items are scored on a 10 cm linear scale.

## Data collection

### Dietary intake

An experienced research dietitian will obtain a comprehensive dietary history from each study participant at baseline and at the completion of each intervention period. A dietary history is a structured interview method consisting of questions regarding habitual food intake. It involves a 24-hour diet recall followed by a food frequency questionnaire to clarify information about usual consumption over the previous month. Usual portion sizes will be obtained in household measures and with the use of photographic aids. The dietitian will review the dietary history with each participant, probing for details on portion sizes and cooking methods in order to improve data accuracy. Dietary AGE content will be estimated from an open source database which lists the AGE concentration of foods using validated analytical techniques [46]. Dietary macro and micronutrient intakes will be estimated using the Foodworks nutrient software program (Xyris Software, NSW, Australia). Each participant will also be randomly contacted by telephone during the study and asked to provide a 24-hour diet recall, and changes in urinary urea excretion will be monitored in order to validate the dietary history data collected [47]. Physical activity will be estimated by asking participants to complete the International Physical Activity Questionnaire (Short Form) prior to and at the completion of experimental and placebo intervention periods [48].

### Anthropometry

Body weight will be measured in participants wearing light clothes without shoes using a digital scale (Seca, Germany) to the nearest 0.1 kg. Height will be measured using a portable stadiometer (Seca, Germany) to the nearest 0.1 cm. BMI is calculated by dividing weight (kg) per height (m) square. Waist circumference will be measured at the midpoint of the lowest rib and iliac crest using a measuring tape to the nearest 0.1 cm. Body composition (total body water, fat mass, fat-free mass) will be determined using Bioelectrical Impedance Analysis (BodyStat-1500, Bodystat, Douglas, Isle of Man, United Kingdom). Ambulatory blood pressure will be measured using an electronic blood pressure machine (Omron Corporation, Kyoto, Japan), with subjects at rest in a seated position. All anthropometric measurements will be conducted in duplicate, with the mean measurement recorded.

### Laboratory investigations

Biological samples will be collected at baseline and at completion of each three-month supplement intervention period and stored at −80°C immediately after collection. Twenty ml of fasting venous blood will be collected from each participant by phlebotomy into a sodium fluoride EDTA tube, a heparin-lined vacuum tube and a clean glass test tube. Twenty-four hour urine collections and morning stool samples will be collected in sterile containers.

Serum, urinary and faecal CML will be measured using a competitive ELISA (AGE-CML ELISA, Microcoat, Penzberg, Germany) [49]. This assay has been validated [50], is specific, and shows no cross-reactivity with other compounds [49]. The within assay and between-assay coefficient of variation are both less than 5%, respectively. Methylglyoxal will be measured by HPLC. Serum total cholesterol and triglyceride concentrations will be determined by enzymatic colorimetric assay (Technicon Instruments, Ltd., New York, N.Y., USA), while HDL cholesterol will be determined enzymatically in the supernatant after precipitation of other lipoproteins with dextran sulphate-magnesium. LDL-cholesterol will be calculated using the Friedewald formula. Plasma glucose levels will be determined by using an automated glucose oxidase method (Glucose analyser 2, Beckman Instruments, Fullerton, California). Insulin will be measured by enzymatic colorimetry (WAKO Pure-Chemical Industries, Osaka, Japan). Insulin Resistance (IR) will be estimated by the homeostasis model assessment (HOMA) index as [FI × (fasting glucose/22.5)], where FI is insulin in microunits per millilitre and fasting glucose is in millimoles per litre [51,52]. HbA1c will be measured by autoanalyser (Roche Diagnostics, Mannheim, Germany). Plasma IL-6, MCP-1, E-selectin, hsCRP, TLR4, glutathione (GSH), and myeloperoxidase will be measured by commercial ELISA kits (Biosource International, Camarillo, CA, USA). Urine 8-isoprostanes will be measured by ELISA (Oxford Biomedical Research, MI, USA).

Stool samples will be homogenised in a blender and stored at −20°C for SCFA analysis. Samples will be thawed and 5 g aliquots placed in Centriprep fluid concentrators, MWCO 30,000 kDa (Amicon Inc., Beverly, MA, USA). Samples will be centrifuged for 30 minutes at 1000 × g, room temperature and supernatants placed in 15 ml polypropylene tubes. 0.3 ml of 25% m-phosphoric acid will be added to each tube, samples will be vortexed and incubated at room temperature for 25 minutes. Samples will be centrifuged at 5000 × g for 15 minutes at room temperature. Supernatants will be decanted and frozen overnight. The following day, samples will be thawed and the pH of each sample adjusted to 6.5 using 4 N KOH. Oxalic acid will be added at a final concentration of 0.03% and SCFA concentrations determined by gas chromatography with use of a Hewlett-Packard 5880A gas chromatograph (Hewlett Packard, Palo Alto, CA, USA) containing an 80/120 Carbopack B-DA/4% Carbowax 20 M column (Supelco Inc., Bellefonte, PA, USA).

Quantitative Real-time PCR will be used to determine faecal concentrations of *Bifidobacterium* spp., *Lactobacillus* spp., *Roseburia* spp., *Faecalbacterium prausnitzii* and *Akkermansia muciniphila*. The primers used will be based on the following 16S rRNA gene sequences: *Bifidobacterium* spp: F-CTCCTGGAAACGGGTGG and R-GGTGTTCTTCCCGATATCTACA [53], *Lactobacillus* spp: F-AGCAGTAGGGAATCTTCCA and R-CACCGCTACACATGGAG [54], *Roseburia* spp: F-CGKACTAGAGTGTCGGAGG and R-GTCATCTAGAGTGTCGGAGG [55], *Faecalbacterium prausnitzii*: F-GGAGGAAGAAGGTCTTCGG and R-AATTCCGCCTACCTCTGCACT [56], and *Akkermansia muciniphila:* F-CAGCACGTGAAGGTGGGGAC and R-CCTTGCGGTTGGCTTCAGAT [57]. PCR amplification and detection will be achieved with an ABI 7300 Real-time PCR system (Applied Biosystems, Foster City, CA, USA) using Mighty Amp for Real-time (SYBR Plus) and Rox Reference Dye (Invitrogen, Carlsbad, CA, USA). Each assay will be performed in duplicate in the same run. The cycle threshold of each sample will then be compared with a standard curve (performed in duplicate) made by diluting genomic DNA (tenfold serial dilution). Prior to isolating the DNA, the cell counts will be determined in culture and expressed as “colony forming units” (CFU). Data will be expressed as log CFU/g of faeces.

### Statistical analysis

Outcome analyses will be undertaken on an intention-to-treat basis. Data will be presented as means ± SD. The Kolmogorov-Smirnov goodness-of-fit test will be used to test for normal distribution, and data not normally distributed will be log-transformed. Correlation analyses will be performed using the Pearson correlation coefficient. Significance of changes during the study will be assessed by comparing change of means between placebo and prebiotic treatment periods by paired sample t-tests. Trial data will be analysed using a linear mixed model design based on repeated measures to account for fixed factors such as treatment sequence (inulin – placebo vs placebo - inulin) and treatment period (intervention 1 vs intervention 2) in addition to participants as a random factor. Significant differences will be defined as a value of P < 0.05 based on two-sided tests. Any differences in physical activity levels, anthropometry, energy or nutrient intake during the course of the trial will be identified using ANOVA. Gastrointestinal symptom data obtained by visual analogue scale will be analysed using the Wilcoxon signed rank test. Effect sizes including 95% confidence intervals will be calculated for all significant outcomes. Data analysis will be performed using SPSS 20.0 software (SPSS, Chicago, IL).

## Discussion

AGEs are derived from both exogenous and endogenous sources, and the rate at which AGEs accumulate in the body is dependent to a large extent on the chronological age, lifestyle and metabolic health of an individual. Smoking cigarettes and consuming foods containing high concentrations of AGEs (and their precursors) increases the accumulation of AGEs from exogenous sources. Endogenous AGE formation is accelerated under conditions of hyperglycaemia, dyslipidaemia and increased oxidative stress, conditions that are common in individuals with diabetes and in those at risk of developing type 2 diabetes (such as those with prediabetes and the metabolic syndrome) [58]. Moreover, in individuals with impaired renal function, urinary AGE excretion may be diminished resulting in a greater accumulation of AGEs in the body [59].

Risk factors for the development of type 2 diabetes include obesity, hypertension and cardiovascular disease; conditions that are commonly associated with unhealthy lifestyles including poor food habits. Restricting the intake of foods high in AGEs might potentially reduce AGE accumulation, but adherence to such diets can be challenging given that foods high in AGEs are very palatable due to their enhanced flavour, colour and aroma [60].

Supplementation of the diet with bifidogenic prebiotic fibres (such as inulin) may reduce or retard the accumulation of AGEs in individuals at risk of developing type 2 diabetes. Prebiotics have been shown to improve and restore optimal microbial balance within the gastrointestinal tract, potentially reducing AGE absorption and/or production by the human host. Preliminary investigations indicate that consuming a high-AGE diet is sufficient to favour the proliferation of potentially pathogenic colonic bacteria over more beneficial species. Consumption of glycated proteins [34], fried meats [61] and toasted wheat flakes [62] encouraged the preferential growth of greater numbers of detrimental gram negative and sulphate-reducing colonic micro-organisms when compared to control diets. Short Chain Fatty Acids (SCFAs) produced as a bacterial by-product of prebiotic fermentation act to lower the intestinal pH, inhibiting the growth of protein-degrading micro-organisms capable of producing potentially toxic metabolites. SCFAs also stimulate colonic smooth muscle contractions, speeding intestinal transit and limiting the time available for protein fermentation and putrefaction to occur in the gut [63]. Therapeutic manipulation of the gut microbiota with prebiotics may restore gut normobiosis and reduce AGE accumulation in humans at risk for type 2 diabetes development by the following mechanisms:

### Maintenance of gut barrier function

Kinetic studies have estimated that up to 30% of dietary AGEs consumed are intestinally absorbed [64]. Under circumstances of increased intestinal permeability, it is likely that greater quantities of dietary AGEs and their reactive dicarbonyl precursors may be able to gain entry into the systemic circulation. Elevated levels of circulating proinflammatory cytokines and ROS frequently observed in individuals with prediabetes are known to compromise tight junctions between cells, disrupting the integrity of the intestinal barrier and enabling the absorption of larger, potentially deleterious compounds [65]. Prebiotic fermentation products may reduce gastrointestinal permeability and as a result limit the absorption of exogenous AGEs.

The SCFAs acetate and butyrate are intricately involved in the maintenance of gut epithelial integrity. Acetate increases colonic blood flow and butyrate is the principal fuel for colonocytes, assisting to optimise epithelial cell health [66]. Butyrate reduces gastrointestinal permeability by enhancing the activation of the peroxisomal proliferator-activated receptor gamma (PPARgamma) gene, a nuclear receptor involved in the attenuation of inflammation in colonic epithelial cells [67]. Butyrate also upregulates the expression of mucin-associated genes important in maintaining the integrity of the intestinal mucosal barrier [68]. Oligofructose supplementation in mice has been shown to increase the expression of zonula and occludin, proteins important in the maintenance of tight junctions between gastrointestinal cells [69], and a dietary inulin intervention reduced markers of intestinal permeability in healthy adult males [70]. Butyrate is a histone deacetylase inhibitor and is likely to promote the transcription of these tight junction proteins. Prebiotic-induced changes in gut microbiota also increase endogenous production of Glucagon-like peptide 2 (GLP-2), which enhances gut barrier function by promoting the proliferation of crypt cells [71,72].

### Reduction of oxidative stress, inflammation and insulin resistance

Increased production of ROS stimulate endogenous AGE formation by oxidising glucose and unsaturated fatty acids to generate reactive dicarbonyls. Experimental drug treatments which attenuate oxidative stress have previously demonstrated reductions in serum AGE levels [73]. Cytokine production at sites of inflammation stimulate immune cell activation of NADPH oxidase (NOX) and production of myeloperoxidase, enzymes involved in the oxidation of amino acids to form AGE precursors [74]. Activated immune cells can also secrete the high-mobility group box 1 (HMGB-1) protein which is capable of binding to RAGE, thereby inducing further inflammation [75].

A high fat diet (independent of the level of obesity in the host) is associated with negative changes in bacterial communities within the colon [76]. In response to a high fat meal, bacterial lipopolysaccharide (LPS) translocates from the intestine into the host circulation, resulting in ‘metabolic endotoxemia’ [77,78]. LPS is a major component of the outer membrane of gram negative bacteria, and is a potent activator of the mammalian immune system. LPS interaction with immune cells stimulates macrophage over-production of ROS, enhances the secretion of pro-inflammatory cytokines, and contributes to weight gain and development of insulin resistance [79,80]. Individuals with type 2 diabetes have been found to possess endotoxemia levels 2-fold higher than people without diabetes [81]. Metabolic endotoxemia also positively correlates with total energy intake and fasting insulin levels in the general population [82]. In mice with high fat diet-induced metabolic endotoxemia, nutritional supplementation of the diet with prebiotics restored intestinal levels of gram positive bacteria (particularly Bifidobacterium species) and subsequently improved glucose tolerance and reduced circulating concentrations of LPS and pro-inflammatory cytokines [83]. Human trials involving dietary prebiotic supplementation have successfully reduced serum LPS levels [45] and markers of lipid peroxidation (a process which generates AGE precursors), possibly through the reduction of ROS production or the direct antioxidant ability of some Bifidobacterium and Lactobacillus bacterial species [84,85].

SCFAs produced as a bacterial by-product of prebiotic fermentation are absorbed into the host circulation, effecting the expression of a wide range of genes in distal tissues associated with cell proliferation, differentiation and apoptosis. SCFAs are ligands for the G-protein coupled receptors (GPRs) GPR41 and GPR43 [86] on immune cells. These receptors are involved in down-regulating inappropriate immune cell production of pro-inflammatory cytokines, chemokines and ROS [87]. The SCFAs acetate, proprionate and butyrate exhibit a variety of anti-inflammatory actions through inhibition of NFκB activation, prevention of LPS-stimulated TNFα production in neutrophils and suppression of cytokine production [31,88-90].

Numerous other immune modulating effects have been observed secondary to gut bacterial activity including the production of anti-inflammatory compounds such as polysaccharide A [91], peptidoglycan [92] and conjugated linoleic acid [93], and the induction of T-regulatory cells [26].

### Promotion of weight reduction

An energy-restricted diet resulting in weight loss has been shown to reduce serum AGE levels in overweight and obese individuals [94]. The consumption of prebiotics in human clinical trials has promoted self-reported satiety [95], weight reduction, reduced production of the orexigenic hormone grehlin and stimulated expression of the appetite-reducing hormone peptide YY (PYY) [96]. Interaction with GPR41 by the SCFAs proprionate and butyrate increases satiety [97], upregulates PYY production and modulates the expression of leptin, a hormone important in controlling energy intake and expenditure [31].

In mice, the selective growth of certain lactobacillus species in the colon reduced body fat storage through the up-regulation of fiaf (fasting induced adipose factor) gene expression and inhibition of lipoprotein lipase (LPL) [98-100]. These findings may have the potential to affect weight reduction in humans and subsequently reduce circulating AGE levels.

### Enhanced antioxidant capacity

Inulin enhances the proliferation of lactic acid producing bacteria capable of synthesising B-group vitamins, some of which have an antioxidant capacity [101]. These vitamins can be utilised by the human host to neutralise ROS. Vitamins B1 and B6 trap the carbonyl groups of highly reactive AGE precursors before they can react with proteins [102]. Some Lactobacillus and Bifidobacterium species are efficient scavengers of the lipid peroxidation product malandialdehyde, protecting the host from excessive accumulation of this toxic AGE precursor [84]. Inulin also exhibits antioxidant properties independent of altering gut bacterial growth and is able to scavenge a number of ROS, which may help to reduce lipid peroxidation in the stomach [103].

The SCFA butyrate, produced as a bacterial by-product of inulin fermentation, has been shown to increase colonic glutathione production [104]. Glutathione is an antioxidant co-factor required for glyoxalase I activity, an enzyme which degrades the AGE precursor methylglyoxyl. Increased production of ROS is also thought to deplete glutathione levels. Through the reduction of oxidative stress, prebiotics may assist in the maintainance or upregulation of the glyoxylase pathway.

### Reduction of hyperglycemia

Both transient and chronic elevations in blood glucose increase endogenous AGE generation. Activation of GPR43 in adipocytes by proprionate inhibits lipolysis and lowers glycemia in healthy individuals [105]. Butyrate has been shown to reverse diet-induced insulin resistance in animal studies [106], possibly by enhancing PPARgamma expression which increases fatty acid oxidation in muscle.

Glucagon-like peptide 1 (GLP-1) is an incretin hormone released from intestinal L-cells in response to consumption of carbohydrates and fats. GLP-1 potentiates glucose-induced insulin secretion, reducing post-prandial blood glucose levels. GLP-1 also enhances satiety and slows gastric emptying. Prebiotic feeding in rats promoted L-cell differentiation in the colon and increased GLP-1 production [107,108], probably through an increase in bacterial production of butyrate.

The consumption of inulin as a dietary supplement may also influence circulating AGE concentrations independently of its prebiotic function. High glycemic index (GI) diets and their resultant hyperglycemic effect have been shown to enhance AGE formation in healthy individuals [109]. Inulin is a soluble fibre which when consumed daily may play a role in the reduction of the GI of the diet. Many soluble fibres are known for their ability to delay gastric emptying and slow the rate of intestinal nutrient absorption, reducing the GI of the carbohydrates in the meal. Regular consumption of prebiotic soluble fibre reduces fasting and post-prandial serum glucose levels in people with impaired glucose tolerance [110] and type 2 diabetes [111].

#### Study strengths

● To our knowledge, this is the first trial to investigate the potential effects of gut bacterial modulation on advanced glycation.

● Random allocation of participants to treatment sequence and intention to treat analysis will ensure study bias is minimised.

● Double-blind crossover placebo-controlled trial.

● Dietary data will be obtained to determine dietary patterns as well as estimates of total energy intake, fat and AGE consumption.

● CML in this study will be measured using a validated ELISA method.

● Dietary AGE consumption will be estimated from an open-source food AGE database, containing AGE values obtained using validated measurement techniques.

#### Study limitations

Type 2 diabetes is a chronic condition which often develops over decades, making it difficult to conduct intervention studies using the presence or absence of diabetes as the primary biological end-point. This study will measure surrogate biochemical markers of early type 2 diabetes pathogenesis, which could be considered a limitation of the trial. Long-term studies will need to be conducted in order to confirm the results of this research.

This study will measure serum CML as an indicator of AGE concentration in blood samples. Multiple other forms of AGEs exist, many of which have not yet been characterised, so the findings of this trial cannot be applied to all members of the AGE family. However, serum CML concentration shows a moderate to high correlation with other known circulating AGEs [39].

#### Applicability of research findings

In Australia, conservative estimates predict that at least 2 million adults will have been diagnosed with type 2 diabetes by 2025 [112]. The burden of disease associated with diabetes has a substantial impact on costs associated directly with health care as well as loss of productivity and decreased quality of life. The widespread consumption of high fat, heat processed foods and the increasing prevalence of obesity in Australia warrant simple interventions including those that prevent AGE-mediated damage. If dietary treatments aimed at altering the gut microbiota prove to be effective strategies for preventing or slowing the development of type 2 diabetes, they could become mainstream therapies for individuals with diabetes risk factors.

## Abbreviations

AGE: Advanced glycation endproduct; sRAGE: Soluble receptor for advanced glycation endproducts; HOMA: Homeostasis model assessment; TAC: Total antioxidant capacity; TNFα: Tumour necrosis factor alpha; IL-6: Interleukin 6; VCAM-1: Vascular cell adhesion molecule 1; RAGE: Receptor for advanced glycation endproducts; CML: Carboxymethyl-lysine; MG: Methylglyoxal; LPS: Lipopolysaccharide; SCFA: Short chain fatty acid; GPR: G-protein coupled receptor; PYY: Peptide YY; GLP-2: Glucagon-like peptide 2; ACE: Angiotensin converting enzyme; DPP-4: Dipeptidyl peptidase 4; Fiaf: Fasting-induced adipocyte factor; ELISA: Enzyme linked immunosorbent assay; NFκB: Nuclear Factor kappa-B; TLR4: Toll-like receptor 4.

## Competing interests

The authors declare that they have no competing interests.

## Authors’ contributions

NJK and MTC designed the study, NJK drafted the manuscript with assistance from MTC, GSS and CMR. All authors read and approved the final manuscript.

## Pre-publication history

The pre-publication history for this paper can be accessed here:

http://www.biomedcentral.com/1472-6823/14/55/prepub

## References

[B1] Henle T Protein-bound advanced glycation endproducts (AGEs) as bioactive amino acid derivatives in foods Amino Acids 2005 29 4 313 322 15997413 10.1007/s00726-005-0200-2

[B2] Brownlee M Biochemistry and molecular cell biology of diabetic complications Nature 2001 414 6865 813 820 11742414 10.1038/414813a

[B3] Folli F Corradi D Fanti P Davalli A Paez A Giaccari A Perego C Muscogiuri G The role of oxidative stress in the pathogenesis of type 2 diabetes mellitus micro- and macrovascular complications: avenues for a mechanistic-based therapeutic approach Curr Diabetes Rev 2011 7 5 313 324 21838680 10.2174/157339911797415585

[B4] Kotani K Sakane N C-reactive protein and reactive oxygen metabolites in subjects with metabolic syndrome J Int Med Res 2012 40 3 1074 1081 22906280 10.1177/147323001204000326

[B5] Thornalley PJ Dicarbonyl intermediates in the maillard reaction Ann N Y Acad Sci 2005 1043 111 117 16037229 10.1196/annals.1333.014

[B6] Avery NC Bailey AJ The effects of the Maillard reaction on the physical properties and cell interactions of collagen Pathol Biol 2006 54 7 387 395 16962252 10.1016/j.patbio.2006.07.005

[B7] Rosca MG Mustata TG Kinter MT Ozdemir AM Kern TS Szweda LI Brownlee M Monnier VM Weiss MF Glycation of mitochondrial proteins from diabetic rat kidney is associated with excess superoxide formation Am J Physiol Renal Physiol 2005 289 2 F420 F430 15814529 10.1152/ajprenal.00415.2004

[B8] Coughlan MT Thorburn DR Penfold SA Laskowski A Harcourt BE Sourris KC Tan AL Fukami K Thallas-Bonke V Nawroth PP Brownlee M Bierhaus A Cooper ME Forbes JM RAGE-induced cytosolic ROS promote mitochondrial superoxide generation in diabetes J Am Soc Nephrol 2009 20 4 742 752 19158353 10.1681/ASN.2008050514 PMC2663823

[B9] Yim MB Yim HS Lee C Kang SO Chock PB Protein glycation: creation of catalytic sites for free radical generation Ann N Y Acad Sci 2001 928 48 53 11795527

[B10] Schmidt AM Hori O Chen JX Li JF Crandall J Zhang J Cao R Yan SD Brett J Stern D Advanced glycation endproducts interacting with their endothelial receptor induce expression of vascular cell adhesion molecule-1 (VCAM-1) in cultured human endothelial cells and in mice: A potential mechanism for the accelerated vasculopathy of diabetes J Clin Invest 1995 96 3 1395 1403 7544803 10.1172/JCI118175 PMC185762

[B11] Morita M Yano S Yamaguchi T Sugimoto T Advanced glycation end products-induced reactive oxygen species generation is partly through NF-kappa B activation in human aortic endothelial cells J Diabetes Complications 2013 27 1 11 15 22944044 10.1016/j.jdiacomp.2012.07.006

[B12] Tahara N Yamagishi S Matsui T Takeuchi M Nitta Y Kodama N Mizoguchi M Imaizumi T Serum levels of advanced glycation end products (AGEs) are independent correlates of insulin resistance in nondiabetic subjects Cardiovasc Ther 2012 30 1 42 48 20626403 10.1111/j.1755-5922.2010.00177.x

[B13] Tan KC Shiu SW Wong Y Tam X Serum advanced glycation end products (AGEs) are associated with insulin resistance Diabetes Metab Res Rev 2011 27 5 488 492 21337488 10.1002/dmrr.1188

[B14] Uribarri J Cai W Sandu O Peppa M Goldberg T Vlassara H Diet-derived advanced glycation end products are major contributors to the body's AGE pool and induce inflammation in healthy subjects Ann N Y Acad Sci 2005 1043 461 466 16037267 10.1196/annals.1333.052

[B15] Fiory F Lombardi A Miele C Giudicelli J Beguinot F Van Obberghen E Methylglyoxal impairs insulin signalling and insulin action on glucose-induced insulin secretion in the pancreatic beta cell line INS-1E Diabetologia 2011 54 11 2941 2952 21861178 10.1007/s00125-011-2280-8

[B16] Coughlan MT Yap FY Tong DC Andrikopoulos S Gasser A Thallas-Bonke V Webster DE Miyazaki J Kay TW Slattery RM Kaye DM Drew BG Kingwell BA Fourlanos S Groop PH Harrison LC Knip M Forbes JM Advanced glycation end products are direct modulators of beta-cell function Diabetes 2011 60 10 2523 2532 21911745 10.2337/db10-1033 PMC3178291

[B17] Birlouez-Aragon I Saavedra G Tessier FJ Galinier A Ait-Ameur L Lacoste F Niamba CN Alt N Somoza V Lecerf JM A diet based on high-heat-treated foods promotes risk factors for diabetes mellitus and cardiovascular diseases Am J Clin Nutr 2010 91 5 1220 1226 20335546 10.3945/ajcn.2009.28737

[B18] Tessier FJ Birlouez-Aragon I Health effects of dietary Maillard reaction products: the results of ICARE and other studies Amino Acids 2012 42 4 1119 1131 20949364 10.1007/s00726-010-0776-z

[B19] Sandu O Song K Cai W Zheng F Uribarri J Vlassara H Insulin resistance and type 2 diabetes in high-fat-fed mice are linked to high glycotoxin intake Diabetes 2005 54 8 2314 2319 16046296 10.2337/diabetes.54.8.2314

[B20] Cai W Ramdas M Zhu L Chen X Striker GE Vlassara H Oral advanced glycation endproducts (AGEs) promote insulin resistance and diabetes by depleting the antioxidant defenses AGE receptor-1 and sirtuin 1 Proc Natl Acad Sci U S A 2012 109 39 15888 15893 22908267 10.1073/pnas.1205847109 PMC3465382

[B21] Uribarri J Cai W Ramdas M Goodman S Pyzik R Chen X Zhu L Striker GE Vlassara H Restriction of advanced glycation end products improves insulin resistance in human type 2 diabetes: potential role of AGER1 and SIRT1 Diabetes Care 2011 34 7 1610 1616 21709297 10.2337/dc11-0091 PMC3120204

[B22] Mark AB Poulsen MW Andersen S Andersen JM Bak MJ Ritz C Holst JJ Nielsen J de Courten B Dragsted LO Bugel S Consumption of a diet low in advanced glycation endproducts for 4 weeks improves insulin sensitivity in overweight women Diabetes Care 2014 37 1 88 95 23959566 10.2337/dc13-0842

[B23] Luevano-Contreras C Garay-Sevilla ME Wrobel K Malacara JM Dietary advanced glycation end products restriction diminishes inflammation markers and oxidative stress in patients with type 2 diabetes mellitus J Clin Biochem Nutr 2013 52 1 22 26 23341693 10.3164/jcbn.12-40 PMC3541414

[B24] Harcourt BE Sourris KC Coughlan MT Walker KZ Dougherty SL Andrikopoulos S Morley AL Thallas-Bonke V Chand V Penfold SA de Courten MP Thomas MC Kingwell BA Bierhaus A Cooper ME Courten B Forbes JM Targeted reduction of advanced glycation improves renal function in obesity Kidney Int 2011 80 2 190 198 21412218 10.1038/ki.2011.57

[B25] Kellow NJ Savige GS Dietary advanced glycation end-product restriction for the attenuation of insulin resistance, oxidative stress and endothelial dysfunction: a systematic review Eur J Clin Nutr 2013 67 3 239 248 23361161 10.1038/ejcn.2012.220

[B26] Round JL Mazmanian SK The gut microbiota shapes intestinal immune responses during health and disease Nat Rev Immunol 2009 9 5 313 323 19343057 10.1038/nri2515 PMC4095778

[B27] Ding S Chi MM Scull BP Rigby R Schwerbrock NM Magness S Jobin C Lund PK High-fat diet: bacteria interactions promote intestinal inflammation which precedes and correlates with obesity and insulin resistance in mouse PLoS One 2010 5 8 e12191 20808947 10.1371/journal.pone.0012191 PMC2922379

[B28] Diamant M Blaak EE de Vos WM Do nutrient-gut-microbiota interactions play a role in human obesity, insulin resistance and type 2 diabetes? Obes Rev 2011 12 4 272 281 20804522 10.1111/j.1467-789X.2010.00797.x

[B29] Backhed F Manchester JK Semenkovich CF Gordon JI Mechanisms underlying the resistance to diet-induced obesity in germ-free mice Proc Natl Acad Sci U S A 2007 104 3 979 984 17210919 10.1073/pnas.0605374104 PMC1764762

[B30] Hill MJ Intestinal flora and endogenous vitamin synthesis Eur J Cancer Prev 1997 6 Suppl 1 S43 S45 9167138 10.1097/00008469-199703001-00009

[B31] Macfarlane GT Macfarlane S Fermentation in the human large intestine: its physiologic consequences and the potential contribution of prebiotics J Clin Gastroenterol 2011 45 Suppl S120 S127 21992950 10.1097/MCG.0b013e31822fecfe

[B32] Wu X Ma C Han L Nawaz M Gao F Zhang X Yu P Zhao C Li L Zhou A Wang J Moore JE Millar BC Xu J Molecular characterisation of the faecal microbiota in patients with type II diabetes Curr Microbiol 2010 61 1 69 78 20087741 10.1007/s00284-010-9582-9

[B33] Larsen N Vogensen FK van den Berg FW Nielsen DS Andreasen AS Pedersen BK Al-Soud WA Sorensen SJ Hansen LH Jakobsen M Gut microbiota in human adults with type 2 diabetes differs from non-diabetic adults PLoS One 2010 5 2 e9085 20140211 10.1371/journal.pone.0009085 PMC2816710

[B34] Mills DJ Tuohy KM Booth J Buck M Crabbe MJ Gibson GR Ames JM Dietary glycated protein modulates the colonic microbiota towards a more detrimental composition in ulcerative colitis patients and non-ulcerative colitis subjects J Appl Microbiol 2008 105 3 706 714 18399977 10.1111/j.1365-2672.2008.03783.x

[B35] Nakamura YK Omaye ST Metabolic diseases and pro- and prebiotics: mechanistic insights Nutr Metab (Lond) 2012 9 1 60 22713169 10.1186/1743-7075-9-60 PMC3464869

[B36] Quigley EM Therapies aimed at the gut microbiota and inflammation: antibiotics, prebiotics, probiotics, synbiotics, anti-inflammatory therapies Gastroenterol Clin North Am 2011 40 1 207 222 21333908 10.1016/j.gtc.2010.12.009

[B37] Delzenne NM Neyrinck AM Backhed F Cani PD Targeting gut microbiota in obesity: effects of prebiotics and probiotics Nat Rev Endocrinol 2011 7 11 639 646 21826100 10.1038/nrendo.2011.126

[B38] Colagiuri SDD Girgis S Colagiuri R National evidence based guideline for case detection and diagnosis of type 2 diabetes 2009 Canberra, Australia: Diabetes Australia and the National Health and Medical Research Council

[B39] Semba RD Arab L Sun K Nicklett EJ Ferrucci L Fat mass is inversely associated with serum carboxymethyl-lysine, an advanced glycation end product, in adults J Nutr 2011 141 9 1726 1730 21775524 10.3945/jn.111.143172 PMC3159057

[B40] Kilhovd BK Juutilainen A Lehto S Ronnemaa T Torjesen PA Hanssen KF Laakso M Increased serum levels of methylglyoxal-derived hydroimidazolone-AGE are associated with increased cardiovascular disease mortality in nondiabetic women Atherosclerosis 2009 205 2 590 594 19185865 10.1016/j.atherosclerosis.2008.12.041

[B41] Lapolla A Piarulli F Sartore G Ceriello A Ragazzi E Reitano R Baccarin L Laverda B Fedele D Advanced glycation end products and antioxidant status in type 2 diabetic patients with and without peripheral artery disease Diabetes Care 2007 30 3 670 676 17327339 10.2337/dc06-1508

[B42] Semba RD Bandinelli S Sun K Guralnik JM Ferrucci L Plasma carboxymethyl-lysine, an advanced glycation end product, and all-cause and cardiovascular disease mortality in older community-dwelling adults J Am Geriatr Soc 2009 57 10 1874 1880 19682127 10.1111/j.1532-5415.2009.02438.x PMC2785105

[B43] Kolida S Meyer D Gibson GR A double-blind placebo-controlled study to establish the bifidogenic dose of inulin in healthy humans Eur J Clin Nutr 2007 61 10 1189 1195 17268410 10.1038/sj.ejcn.1602636

[B44] Causey JL Effects of dietary inulin on serum lipids, blood glucose and the gastrointestinal environment in hypercholesterolemic men Nutr Res 2000 20 2 191 201

[B45] Lecerf JM Depeint F Clerc E Dugenet Y Niamba CN Rhazi L Cayzeele A Abdelnour G Jaruga A Younes H Jacobs H Lambrey G Abdelnour AM Pouillart PR Xylo-oligosaccharide (XOS) in combination with inulin modulates both the intestinal environment and immune status in healthy subjects, while XOS alone only shows prebiotic properties Br J Nutr 2012 108 10 1847 1858 22264499 10.1017/S0007114511007252

[B46] Hull GLJ Ames JM Cuskelly GJ NEpsilon-(carboxymethyl)lysine content of foods commonly consumed in a Western style diet Food Chem 2012 131 170 174

[B47] Bihuniak JD Simpson CA Sullivan RR Caseria DM Kerstetter JE Insogna KL Dietary protein-induced increases in urinary calcium are accompanied by similar increases in urinary nitrogen and urinary urea: a controlled clinical trial J Acad Nutr Diet 2013 113 3 447 451 23438496 10.1016/j.jand.2012.11.002 PMC5868414

[B48] Craig CL Marshall AL Sjostrom M Bauman AE Booth ML Ainsworth BE Pratt M Ekelund U Yngve A Sallis JF Oja P International physical activity questionnaire: 12-country reliability and validity Med Sci Sports Exerc 2003 35 8 1381 1395 12900694 10.1249/01.MSS.0000078924.61453.FB

[B49] Boehm BO Schilling S Rosinger S Lang GE Lang GK Kientsch-Engel R Stahl P Elevated serum levels of N(epsilon)-carboxymethyl-lysine, an advanced glycation end product, are associated with proliferative diabetic retinopathy and macular oedema Diabetologia 2004 47 8 1376 1379 15258735 10.1007/s00125-004-1455-y

[B50] Zhang X Frischmann M Kientsch-Engel R Steinmann K Stopper H Niwa T Pischetsrieder M Two immunochemical assays to measure advanced glycation end-products in serum from dialysis patients Clin Chem Lab Med 2005 43 5 503 511 15899672 10.1515/CCLM.2005.089

[B51] Matthews DR Hosker JP Rudenski AS Naylor BA Treacher DF Turner RC Homeostasis model assessment: insulin resistance and beta-cell function from fasting plasma glucose and insulin concentrations in man Diabetologia 1985 28 7 412 419 3899825 10.1007/BF00280883

[B52] Bonora E Targher G Alberiche M Bonadonna RC Saggiani F Zenere MB Monauni T Muggeo M Homeostasis model assessment closely mirrors the glucose clamp technique in the assessment of insulin sensitivity: studies in subjects with various degrees of glucose tolerance and insulin sensitivity Diabetes Care 2000 23 1 57 63 10857969 10.2337/diacare.23.1.57

[B53] Matsuki T Watanabe K Fujimoto J Miyamoto Y Takada T Matsumoto K Oyaizu H Tanaka R Development of 16S rRNA-gene-targeted group-specific primers for the detection and identification of predominant bacteria in human feces Appl Environ Microbiol 2002 68 11 5445 5451 12406736 10.1128/AEM.68.11.5445-5451.2002 PMC129894

[B54] Dewulf EM Cani PD Claus SP Fuentes S Puylaert PG Neyrinck AM Bindels LB de Vos WM Gibson GR Thissen JP Delzenne NM Insight into the prebiotic concept: lessons from an exploratory, double blind intervention study with inulin-type fructans in obese women Gut 2013 62 8 1112 1121 23135760 10.1136/gutjnl-2012-303304 PMC3711491

[B55] Ramirez-Farias C Slezak K Fuller Z Duncan A Holtrop G Louis P Effect of inulin on the human gut microbiota: stimulation of Bifidobacterium adolescentis and Faecalibacterium prausnitzii Br J Nutr 2009 101 4 541 550 18590586 10.1017/S0007114508019880

[B56] Wang RF Cao WW Cerniglia CE PCR detection and quantitation of predominant anaerobic bacteria in human and animal fecal samples Appl Environ Microbiol 1996 62 4 1242 1247 8919784 10.1128/aem.62.4.1242-1247.1996 PMC167889

[B57] Everard A Belzer C Geurts L Ouwerkerk JP Druart C Bindels LB Guiot Y Derrien M Muccioli GG Delzenne NM de Vos WM Cani PD Cross-talk between Akkermansia muciniphila and intestinal epithelium controls diet-induced obesity Proc Natl Acad Sci U S A 2013 110 22 9066 9071 23671105 10.1073/pnas.1219451110 PMC3670398

[B58] Sebekova K Boor P Valachovicova M Blazicek P Parrak V Babinska K Heidland A Krajcovicova-Kudlackova M Association of metabolic syndrome risk factors with selected markers of oxidative status and microinflammation in healthy omnivores and vegetarians Mol Nutr Food Res 2006 50 9 858 868 16917805 10.1002/mnfr.200500170

[B59] Vlassara H Uribarri J Cai W Striker G Advanced glycation end product homeostasis: exogenous oxidants and innate defenses Ann N Y Acad Sci 2008 1126 46 52 18448795 10.1196/annals.1433.055

[B60] Goldberg T Cai W Peppa M Dardaine V Baliga BS Uribarri J Vlassara H Advanced glycoxidation end products in commonly consumed foods J Am Diet Assoc 2004 104 8 1287 1291 15281050 10.1016/j.jada.2004.05.214

[B61] Shen Q Chen YA Tuohy KM A comparative in vitro investigation into the effects of cooked meats on the human faecal microbiota Anaerobe 2010 16 6 572 577 20934523 10.1016/j.anaerobe.2010.09.007

[B62] Connolly ML Lovegrove JA Tuohy KM In vitro fermentation characteristics of whole grain wheat flakes and the effect of toasting on prebiotic potential J Med Food 2012 15 1 33 43 21877952 10.1089/jmf.2011.0006

[B63] De Preter V Hamer HM Windey K Verbeke K The impact of pre- and/or probiotics on human colonic metabolism: does it affect human health? Mol Nutr Food Res 2011 55 1 46 57 21207512 10.1002/mnfr.201000451

[B64] Faist V Erbersdobler HF Metabolic transit and in vivo effects of melanoidins and precursor compounds deriving from the Maillard reaction Ann Nutr Metab 2001 45 1 1 12 11244181 10.1159/000046699

[B65] Rapin JR Wiernsperger N Possible links between intestinal permeability and food processing: A potential therapeutic niche for glutamine Clinics (Sao Paulo) 2010 65 6 635 643 20613941 10.1590/S1807-59322010000600012 PMC2898551

[B66] Scheppach W Effects of short chain fatty acids on gut morphology and function Gut 1994 35 1 Suppl S35 S38 8125387 10.1136/gut.35.1_suppl.s35 PMC1378144

[B67] Eun CS Han DS Lee SH Paik CH Chung YW Lee J Hahm JS Attenuation of colonic inflammation by PPARgamma in intestinal epithelial cells: effect on Toll-like receptor pathway Dig Dis Sci 2006 51 4 693 697 16614990 10.1007/s10620-006-3193-0

[B68] Canani RB Costanzo MD Leone L Pedata M Meli R Calignano A Potential beneficial effects of butyrate in intestinal and extraintestinal diseases World J Gastroenterol 2011 17 12 1519 1528 21472114 10.3748/wjg.v17.i12.1519 PMC3070119

[B69] Delzenne NM Cani PD Interaction between obesity and the gut microbiota: relevance in nutrition Annu Rev Nutr 2011 31 15 31 21568707 10.1146/annurev-nutr-072610-145146

[B70] Russo F Linsalata M Clemente C Chiloiro M Orlando A Marconi E Chimienti G Riezzo G Inulin-enriched pasta improves intestinal permeability and modifies the circulating levels of zonulin and glucagon-like peptide 2 in healthy young volunteers Nutr Res 2012 32 12 940 946 23244539 10.1016/j.nutres.2012.09.010

[B71] Cani PD Possemiers S Van de Wiele T Guiot Y Everard A Rottier O Geurts L Naslain D Neyrinck A Lambert DM Muccioli GG Delzenne NM Changes in gut microbiota control inflammation in obese mice through a mechanism involving GLP-2-driven improvement of gut permeability Gut 2009 58 8 1091 1103 19240062 10.1136/gut.2008.165886 PMC2702831

[B72] Cani PD Crosstalk between the gut microbiota and the endocannabinoid system: impact on the gut barrier function and the adipose tissue Clin Microbiol Infect 2012 18 Suppl 4 50 53 22647050 10.1111/j.1469-0691.2012.03866.x

[B73] Schalkwijk CG Miyata T Early- and advanced non-enzymatic glycation in diabetic vascular complications: the search for therapeutics Amino Acids 2012 42 4 1193 1204 20960212 10.1007/s00726-010-0779-9 PMC3296013

[B74] Anderson MM Heinecke JW Production of N(epsilon)-(carboxymethyl)lysine is impaired in mice deficient in NADPH oxidase: a role for phagocyte-derived oxidants in the formation of advanced glycation end products during inflammation Diabetes 2003 52 8 2137 2143 12882933 10.2337/diabetes.52.8.2137

[B75] Huebschmann AG Regensteiner JG Vlassara H Reusch JE Diabetes and advanced glycoxidation end products Diabetes Care 2006 29 6 1420 1432 16732039 10.2337/dc05-2096

[B76] Hildebrandt MA Hoffmann C Sherrill-Mix SA Keilbaugh SA Hamady M Chen YY Knight R Ahima RS Bushman F Wu GD High-fat diet determines the composition of the murine gut microbiome independently of obesity Gastroenterology 2009 137 5 1716 1724 e1711-1712 19706296 10.1053/j.gastro.2009.08.042 PMC2770164

[B77] Vrieze A Holleman F Zoetendal EG de Vos WM Hoekstra JB Nieuwdorp M The environment within: how gut microbiota may influence metabolism and body composition Diabetologia 2010 53 4 606 613 20101384 10.1007/s00125-010-1662-7 PMC2830587

[B78] Esteve E Ricart W Fernandez-Real JM Gut microbiota interactions with obesity, insulin resistance and type 2 diabetes: did gut microbiote co-evolve with insulin resistance? Curr Opin Clin Nutr Metab Care 2011 14 5 483 490 21681087 10.1097/MCO.0b013e328348c06d

[B79] Cani PD Amar J Iglesias MA Poggi M Knauf C Bastelica D Neyrinck AM Fava F Tuohy KM Chabo C Waget A Delmee E Cousin B Sulpice T Chamontin B Ferrieres J Tanti JF Gibson GR Casteilla L Delzenne NM Alessi MC Burcelin R Metabolic endotoxemia initiates obesity and insulin resistance Diabetes 2007 56 7 1761 1772 17456850 10.2337/db06-1491

[B80] Priebe MG Wang H Weening D Schepers M Preston T Vonk RJ Factors related to colonic fermentation of nondigestible carbohydrates of a previous evening meal increase tissue glucose uptake and moderate glucose-associated inflammation Am J Clin Nutr 2010 91 1 90 97 19889821 10.3945/ajcn.2009.28521

[B81] Creely SJ McTernan PG Kusminski CM Fisher FM Da Silva NF Khanolkar M Evans M Harte AL Kumar S Lipopolysaccharide activates an innate immune system response in human adipose tissue in obesity and type 2 diabetes Am J Physiol Endocrinol Metab 2007 292 3 E740 E747 17090751 10.1152/ajpendo.00302.2006

[B82] Amar J Energy intake is associated with endotoxemia induces adipose inflammation and insulin resistance in humans Diabetes 2010 59 9 10.2337/db09-0367 PMC2797919 19794059

[B83] Cani PD Neyrinck AM Fava F Knauf C Burcelin RG Tuohy KM Gibson GR Delzenne NM Selective increases of bifidobacteria in gut microflora improve high-fat-diet-induced diabetes in mice through a mechanism associated with endotoxaemia Diabetologia 2007 50 11 2374 2383 17823788 10.1007/s00125-007-0791-0

[B84] Lin MY Yen CL Inhibition of lipid peroxidation by Lactobacillus acidophilus and Bifidobacterium longum J Agric Food Chem 1999 47 9 3661 3664 10552700 10.1021/jf981235l

[B85] Yen CH Kuo YW Tseng YH Lee MC Chen HL Beneficial effects of fructo-oligosaccharides supplementation on fecal bifidobacteria and index of peroxidation status in constipated nursing-home residents–a placebo-controlled, diet-controlled trial Nutrition 2011 27 3 323 328 20579847 10.1016/j.nut.2010.02.009

[B86] Maslowski KM Vieira AT Ng A Kranich J Sierro F Yu D Schilter HC Rolph MS Mackay F Artis D Xavier RJ Teixeira MM Mackay CR Regulation of inflammatory responses by gut microbiota and chemoattractant receptor GPR43 Nature 2009 461 7268 1282 1286 19865172 10.1038/nature08530 PMC3256734

[B87] Vinolo MA Rodrigues HG Nachbar RT Curi R Regulation of inflammation by short chain fatty acids Nutrients 2011 3 10 858 876 22254083 10.3390/nu3100858 PMC3257741

[B88] Lewis K Lutgendorff F Phan V Soderholm JD Sherman PM McKay DM Enhanced translocation of bacteria across metabolically stressed epithelia is reduced by butyrate Inflamm Bowel Dis 2010 16 7 1138 1148 20024905 10.1002/ibd.21177

[B89] Erridge C Attina T Spickett CM Webb DJ A high-fat meal induces low-grade endotoxemia: evidence of a novel mechanism of postprandial inflammation Am J Clin Nutr 2007 86 5 1286 1292 17991637 10.1093/ajcn/86.5.1286

[B90] Krogh-Madsen R Plomgaard P Akerstrom T Moller K Schmitz O Pedersen BK Effect of short-term intralipid infusion on the immune response during low-dose endotoxemia in humans Am J Physiol Endocrinol Metab 2008 294 2 E371 E379 18056792 10.1152/ajpendo.00507.2007

[B91] Macia L Thorburn AN Binge LC Marino E Rogers KE Maslowski KM Vieira AT Kranich J Mackay CR Microbial influences on epithelial integrity and immune function as a basis for inflammatory diseases Immunol Rev 2012 245 1 164 176 22168419 10.1111/j.1600-065X.2011.01080.x

[B92] Maslowski KM Mackay CR Diet, gut microbiota and immune responses Nat Immunol 2011 12 1 5 9 21169997 10.1038/ni0111-5

[B93] Musso G Gambino R Cassader M Interactions between gut microbiota and host metabolism predisposing to obesity and diabetes Annu Rev Med 2011 62 361 380 21226616 10.1146/annurev-med-012510-175505

[B94] Gugliucci A Kotani K Taing J Matsuoka Y Sano Y Yoshimura M Egawa K Horikawa C Kitagawa Y Kiso Y Kimura S Sakane N Short-term low calorie diet intervention reduces serum advanced glycation end products in healthy overweight or obese adults Ann Nutr Metab 2009 54 3 197 201 19420913 10.1159/000217817

[B95] Cani PD Joly E Horsmans Y Delzenne NM Oligofructose promotes satiety in healthy human: a pilot study Eur J Clin Nutr 2006 60 5 567 572 16340949 10.1038/sj.ejcn.1602350

[B96] Parnell JA Reimer RA Weight loss during oligofructose supplementation is associated with decreased ghrelin and increased peptide YY in overweight and obese adults Am J Clin Nutr 2009 89 6 1751 1759 19386741 10.3945/ajcn.2009.27465 PMC3827013

[B97] Ruijschop RBA te Giffela MC Satiety effects of a dairy beverage fermented with propionic acid bacteria Int Dairy J 2008 18 5

[B98] Backhed F Ding H Wang T Hooper LV Koh GY Nagy A Semenkovich CF Gordon JI The gut microbiota as an environmental factor that regulates fat storage Proc Natl Acad Sci U S A 2004 101 44 15718 15723 15505215 10.1073/pnas.0407076101 PMC524219

[B99] Aronsson L Huang Y Parini P Korach-Andre M Hakansson J Gustafsson JA Pettersson S Arulampalam V Rafter J Decreased fat storage by Lactobacillus paracasei is associated with increased levels of angiopoietin-like 4 protein (ANGPTL4) PLoS One 2010 5 9 doi:10.1371/journal.pone.0013087 10.1371/journal.pone.0013087 PMC2948012 20927337

[B100] Dewulf EM Cani PD Neyrinck AM Possemiers S Van Holle A Muccioli GG Deldicque L Bindels LB Pachikian BD Sohet FM Mignolet E Francaux M Larondelle Y Delzenne NM Inulin-type fructans with prebiotic properties counteract GPR43 overexpression and PPARgamma-related adipogenesis in the white adipose tissue of high-fat diet-fed mice J Nutr Biochem 2011 22 8 712 722 21115338 10.1016/j.jnutbio.2010.05.009

[B101] Vyas U Ranganathan N Probiotics, prebiotics, and synbiotics: gut and beyond Gastroenterology Res Pract 2012 2012 872716 10.1155/2012/872716 PMC3459241 23049548

[B102] Mehta R Shangari N O'Brien PJ Preventing cell death induced by carbonyl stress, oxidative stress or mitochondrial toxins with vitamin B anti-AGE agents Mol Nutr Food Res 2008 52 3 379 385 17918169 10.1002/mnfr.200600190

[B103] Stoyanova S Geuns J Hideg E Van Den Ende W The food additives inulin and stevioside counteract oxidative stress Int J Food Sci Nutr 2011 62 3 207 214 21043580 10.3109/09637486.2010.523416

[B104] Wu WT Chen HL Effects of konjac glucomannan on putative risk factors for colon carcinogenesis in rats fed a high-fat diet J Agric Food Chem 2011 59 3 989 994 21208006 10.1021/jf103532x

[B105] Todesco T Rao AV Bosello O Jenkins DJ Propionate lowers blood glucose and alters lipid metabolism in healthy subjects Am J Clin Nutr 1991 54 5 860 865 1951157 10.1093/ajcn/54.5.860

[B106] Gao Z Yin J Zhang J Ward RE Martin RJ Lefevre M Cefalu WT Ye J Butyrate improves insulin sensitivity and increases energy expenditure in mice Diabetes 2009 58 7 1509 1517 19366864 10.2337/db08-1637 PMC2699871

[B107] Cani PD Hoste S Guiot Y Delzenne NM Dietary non-digestible carbohydrates promote L-cell differentiation in the proximal colon of rats Br J Nutr 2007 98 1 32 37 17367575 10.1017/S0007114507691648

[B108] Cani PD Knauf C Iglesias MA Drucker DJ Delzenne NM Burcelin R Improvement of glucose tolerance and hepatic insulin sensitivity by oligofructose requires a functional glucagon-like peptide 1 receptor Diabetes 2006 55 5 1484 1490 16644709 10.2337/db05-1360

[B109] Uchiki T Weikel KA Jiao W Shang F Caceres A Pawlak D Handa JT Brownlee M Nagaraj R Taylor A Glycation-altered proteolysis as a pathobiologic mechanism that links dietary glycemic index, aging, and age-related disease (in nondiabetics) Aging Cell 2012 11 1 1 13 21967227 10.1111/j.1474-9726.2011.00752.x PMC3257376

[B110] Garcia AL Otto B Reich SC Weickert MO Steiniger J Machowetz A Rudovich NN Mohlig M Katz N Speth M Meuser F Doerfer J Zunft HJ Pfeiffer AH Koebnick C Arabinoxylan consumption decreases postprandial serum glucose, serum insulin and plasma total ghrelin response in subjects with impaired glucose tolerance Eur J Clin Nutr 2007 61 3 334 341 16988651 10.1038/sj.ejcn.1602525

[B111] Lu ZX Walker KZ Muir JG O'Dea K Arabinoxylan fibre improves metabolic control in people with Type II diabetes Eur J Clin Nutr 2004 58 4 621 628 15042130 10.1038/sj.ejcn.1601857

[B112] Magliano DJ Peeters A Vos T Sicree R Shaw J Sindall C Haby M Begg SJ Zimmet PZ Projecting the burden of diabetes in Australia–what is the size of the matter? Aust N Z J Public Health 2009 33 6 540 543 20078571 10.1111/j.1753-6405.2009.00450.x

